# Neurotropin alleviates rat osteocarcinoma pain via P_2_X_3_ receptor activation in the midbrain periaqueductal gray 

**DOI:** 10.22038/IJBMS.2021.57965.12904

**Published:** 2021-10

**Authors:** Xingfeng Liu, Jingxin He, Zhi Xiao

**Affiliations:** 1 Key Laboratory of Brain Science, Zunyi Medical University, No.6 West Xuefu Road, Xinpu District, Zunyi, Guizhou, China; 2 Graduate School, Zunyi Medical University, No.6 West Xuefu Road, Xinpu District, Zunyi, Guizhou, China

**Keywords:** A-317491, Bone, Neurotropin, Osteocarcinoma pain, P_2_X_3 _receptor, Periaqueductal gray

## Abstract

**Objective(s):**

Clinically effective analgesia treatment for patients afflicted with osteocarcinoma lessens the intensity of pain. The midbrain periaqueductal gray (PAG) plays a critical role in pain modulation, and activation of P_2_X_3_ receptors in this region mediates pain processing. Neurotropin is a small molecule drug used for analgesic treatment of a number of chronic pain conditions. The present study aims at determining whether P_2_X_3_ receptor activation in PAG is responsible for the analgesic effect of neurotropin in rats with osteocarcinoma pain.

**Materials and Methods:**

The tibia of female Sprague-Dawley rats was inoculated with breast carcinoma cells to establish the osteocarcinoma pain model. The effects of intraperitoneal injection of 6, 12, and 18 neurotropin units (NU)/kg on pain threshold and receptor expression of P_2_X_3 _in the ventrolateral PAG (vlPAG) were assessed. The P_2_X_3_ receptor antagonist A-317491 (1.5 nmol/0.3 µl) was administered into vlPAG with a high-dose neurotropin (18 NU/kg) to determine the role of this receptor in the analgesic effect.

**Results:**

The pain thresholds of the rats with osteocarcinoma pain continuously decreased, whereas P_2_X_3_ receptor expression in vlPAG only slightly increased after osteocarcinoma cell inoculation. Neurotropin substantially elevated the pain threshold and P_2_X_3_ receptor expression in vlPAG in a dose-dependent manner. A-317491 microinjection into vlPAG significantly reduced the analgesic effects of neurotropin in the rats with osteocarcinoma pain.

**Conclusion:**

Through these findings, it is shown that vlPAG P_2_X_3_ receptor activation participates in neurotropin-mediated analgesia mechanism in osteocarcinoma pain.

## Introduction

Despite tremendous improvements in the early detection and treatment of cancer, advances in the clinical management of cancer pain have largely fallen short of what is ideal or required. Many tumors, such as breast and prostate cancer, tend to metastasize to bone, resulting in osteocarcinoma pain, which is a substantial cause of pain and suffering in advanced cancer (1). The three-step analgesic ladder therapy is the primary treatment for cancer pain as adjuvant therapies, such as surgery, glucocorticoid therapy, and radiation therapy, have inadequate pain-relief potency and/or obvious side effects (2, 3). A longstanding yet largely unfulfilled goal is finding effective analgesic strategies with minimal side effects to improve the quality of life in patients suffering from osteocarcinoma pain.

Extracellular adenosine 5′-triphosphate (ATP) is a bona fide rapid neurotransmitter that mediates synaptic transmission by directly binding to P2X/Y receptors (4). Several purine receptor subtypes have been reported to be involved in the formation and transmission of pain, and its maintenance (5). The P_2_X_3_ receptor is the most thoroughly studied purine receptor subtype, and its expression and functional changes are implicated in multiple pain phenotypes. In rat models with inflammatory and neuropathic pain, P_2_X_3_ receptor expression is observed to be altered in peripheral neurons, dorsal root ganglia, and superficial laminae of the dorsal horn (6, 7). Studies specifically related to osteocarcinoma pain have begun to elucidate the role of the purinergic signaling pathway, and they have implicated P_2_X_3_ receptor activation in the early development of osteocarcinoma pain (8). At peripheral sites, the P_2_X_3_ receptor is expressed in epidermal nerve fibers that innervate the tumor-infiltrating bone or trigeminal ganglions, and ATP released by cancer cells is involved in osteocarcinoma pain mechanisms through P_2_X_3_ receptor signaling (9, 10). Wu and colleagues (8) observed that lysophosphatidic acid and its receptor subtype 1 affect P_2_X_3_ receptor expression and function through the RHO-ROCK signaling pathway in dorsal root ganglions and regulate the development of mechanical and spontaneous pain in rats with osteocarcinoma pain. However, the relationship between P_2_X_3_ receptor activation and osteocarcinoma pain mechanisms in supraspinal structures has been rarely reported.

The midbrain periaqueductal gray (PAG) is a critical neural circuit for pain modulation, particularly its ventrolateral region (vlPAG) and its descending projections to the rostral ventromedial medulla and the spinal cord (11-13). In our previous work, we found that the pain thresholds of rats with neuropathic pain decreased, whereas P_2_X_3_ receptor expression in the lateral PAG (lPAG) only mildly increased. Intra-lPAG injection of a P_2_X_3_ receptor agonist (α, β-meATP) could ameliorate the neuropathic pain symptoms in these rats. P_2_X_3_ receptors antagonist A-317491 was used to pretreat lPAG reduced the antinociceptive effect produced by α, β-meATP (14). According to these findings, it is suggested that activation of the P_2_X_3_ receptor in lPAG promotes the efficiency of analgesic signaling and that this receptor is responsible, at least in part, for the analgesic effect on neuropathic pain.

Neurotropin is an extract prepared from rabbit skin following vaccinia virus inoculation. It has been widely used across Asia for the treatment of neuropathic, inflammatory, and other types of chronic pain (15). Despite its widespread use, detailed mechanistic insights into the analgesic effects of neurotropin are lacking, particularly as it relates to the purine signaling pathway. Accordingly, the current study aims at determining the analgesic effect of neurotropin in osteocarcinoma pain, and whether P_2_X_3_ receptor activation in vlPAG is responsible for mediating the analgesic effects. The results show that neurotropin could be considered a potential therapeutic agent for osteocarcinoma pain as it activates the purinergic P_2_X_3_ receptors in vlPAG.

## Materials and Methods

***Chemicals***

Neurotropin was bought from Osaka of Japan, RPMI-1640 medium was obtained from Sangon Biotech (China), isoflurane was acquired from RWD Life Science (China ), rabbit anti-rat P_2_X_3_ polyclonal antiserum was procured from Abcam Corporation (USA), rat monoclonal anti-β-actin was purchased from Millipore Corporation ( USA), and A-317491 was provided by Sigma-Aldrich (CAS number A2979, PubChem Substance ID: 329770806) and dissolved in sterile physiological saline solution with the concentration of 5 µmol/ml.

***Animals***

Female Sprague Dawley rats with a weight of 210±10 g were acquired from Changsha Tianqin Biotechnology Co. Ltd. (Changsha of China). Five rats were fed in each cage and subjected to a 12 hr light/dark cycle (8 am-–8 pm). The ambient temperature was kept at 25±2 °C, and the relative humidity was 55± 5%. The rats accessed food and water freely. Baseline sensitivity to pain induced by mechanical and thermal stimuli was assessed for all experimental animals on the day before the experiment. Only rats who had normal baseline responses to mechanical and thermal pain stimulation were used in this experiment. All guiding principles and general directions of the Ethical Committee of the International Association for the Study of Pain and the Ethics Committee of Animal Care of Zunyi Medical University (2019:2-036) were followed for experimental procedures in this study (16, 17). The number of animal experimental procedures was minimized.

***Cancer cell preparation***

The mammary cancer cell line of the rat (SHZ-88) was procured from the Cell Resource Center of Shanghai Institutes for Biological Sciences and was cultured in RPMI-1640 medium with 5% CO_2_ at 37 °C. The cells were passaged once weekly with fresh media added every two days. The cells were trypsinized with 0.25 % trypsin (5-8 min), and the reaction was stopped by 10% fetal bovine serum. Following trypsinization, the cells had been centrifuged for 3 min at 1000 rpm. The pellet resuspended in RPMI-1640 medium reached the concentration of 10^7 ^cells/0.5 ml finally. Cell counts were obtained with a hemocytometer. 

***Establishment of a rat model of osteocarcinoma pain***

An osteocarcinoma pain model was established in the rats as described previously (18). Briefly, a deep and stable plane of anesthesia was established in the rats with isoflurane (3% to 4% induction and 2% maintenance), and they were placed on a thermally-regulated mat in a prone position. The medial of the proximal tibia was carefully exposed with a 1.5 cm incision to minimize muscle and nerve damage, and SHZ-88 carcinoma cells (10^7^ cells/0.5 ml, 20 μl) were slowly injected into the tibia medullary cavity. Following syringe retraction, the injection site was sealed with sterile bone wax immediately to prevent cancer cell extravasation. Penicillin powder was dispensed to the incision to prevent infection topically. The sham-osteocarcinoma pain group was treated similarly, with the exception of the cancer cells being heat-shocked at 90 °C for 20 min prior to injection. Inoculation by the carcinoma cells occurred on day 0. 

***Bone radiography***

Bone destruction was assessed 21 days after cancer cell inoculation via X-ray detection. Rats were anesthetized by intraperitoneal injection with 4% chloral hydrate (10 ml/kg, IP). The hind limb was exposed to an X-ray source for 0.32 sec at 60 kV and 7 mA (Kodak, CS2200). Destruction of each tibia was assessed by trained radiologists in a double-blind fashion.

***Cannula implantation and microinjection procedures***

Anesthesia was induced in the rats with 3-4% isoflurane followed by a 2% maintenance dose, then mounted on a stereotaxic frame (68025, RWD Life Science Co., Ltd, Shenzhen, China). Following location of the bregma after skull exposure, a metal guide cannula (outside diameter of 0.48 mm and inside diameter of 0.34 mm) was anchored to the skull with dental zinc cement and jewelers’ screws. Stereotaxic coordinates for vlPAG were 5.5 mm ventral to skull surface, 7.90 mm at the back of the bregma, and 0.80 mm on the side of the midline. During the procedure, a dummy cannula was inserted into the guide cannula to reduce the incidence of occlusion. Rats were then removed from the stereotaxic apparatus, and placed in a temperature-controlled cage to prevent hypothermia until recovery. The rats were monitored daily for signs of movement defects for four days prior to experimentation, and any rats exhibiting functional deficits resulting from the operation, such as ataxia, nystagmus, or nausea, were excluded. On the microinjection procedure day, the rats were sent to the laboratory, then they were kept undisturbed for one hour before administrating the drug. For the cannulation procedure, an injection cannula was inserted (outside diameter 0.3 mm; 0.5 mm longer than the guide cannula) into the guide cannula and the injector connected to a miniature syringe pump. 

0.3 μl drug solution was infused slowly over 3 min, and the injector remained in place for an additional minute to ensure complete drug diffusion. To initiate euthanasia, the rats were anesthetized with 2% isofluorane deeply and the hearts were perfused with physiological saline solution (0.9% NaCl) before a 4% paraformaldehyde solution. Visual confirmation of the cannula tip was obtained by 0.1 μl of 2% Evans blue injection through the microinjection cannula when the experiment was completed. Sites of administration in the brain were verified histologically and plotted on coronal maps as previously described (19). The rats who had a confirmed microinjection other than vlPAG were excluded from the statistics.

***Mechanical withdrawal threshold (MWT)***

Mechanical withdrawal thresholds (MWTs) were assessed by placing rats in a separated transparent plexiglass house (18 cm × 12 cm × 12 cm) with a wire mesh floor, which provided a quiet environment. MWT was determined five times in each hind paw, and average values were used. Mechanical allodynia was assessed as previously reported (20). Briefly, hind paw flexion reflex was triggered with an electronic *von Frey* algometer containing a polypropylene tip with a contact area of 0.5 mm^2^. Gradually increasing pressure was applied to the tip vertically to the plantar surface’s central area on the hind paw, and the endpoint was featured by quickly removing the hind paw (the animal actively removed the whole paw upon contact with the tip of the algometer, either by licking its paw, shaking it with high amplitude, or by squeaking in response to the stimuli). 

***Thermal withdrawal latency (TWL)***

In accordance with a previous work (21), heat hypersensitivity was evaluated using a radiant heat source to assess thermal withdrawal latency (TWL). The rats were put in a clean plastic chamber to be acclimatized until they settled down. Subsequently, radiant heat of 52 ± 0.2 °C (50 W, 8 V bulb) was applied to the plantar surface of the hind paw. The latency period was the time recorded between beam initiation and functional pain response (e.g., hind paw lifting or licking, flicking, jumping). Tissue damage was prevented by limiting the cut-off to 60 sec. Measurements were made in each hind paw three times at intervals of 3 min. The mean value was considered as the TWL value. 

***Immunohistochemistry***

The number of P_2_X_3 _receptor-positive cells in the vlPAG was determined via immunohistochemistry. Following euthanasia, rats were decapitated and their brains were post-fixed in 4% paraformaldehyde then transferred to 30% sucrose and cryoprotected. PAGs at a thickness of 25 μm were continuously cut along the axis with a cryostat microtome. Immunohistochemical analysis of the PAG was performed randomly in one out of every five to six sections. The sections were incubated with 3% H_2_O_2_ in 10 mM PBS, before blocking using 3% bovine serum albumin (BSA) in 100 mM PBS. After incubating at 37 °C for 1 hr, the sections were stained for 24 hr at 4 °C with primary antibody (1:400 rabbit anti-rat P_2_X_3_ polyclonal antiserum), followed by secondary antibody (5% goat anti-rabbit serum) (Beijing Zhongshan Biotech Co., China) for an additional 24 hr at 4 °C. Finally, sections were mounted on glass slides, dehydrated through ascending series of alternating ethanol and xylene solutions, and topped with a coverslip. P_2_X_3_ receptor staining in whole-mount preparations of the vlPAG sections was analyzed with the Leica Q500IW image analysis system as previously described (22). Immunoreactive positive neuron bodies in vlPAG were counted per visual field in a 20× objective (total magnification 200×). Only the cells which have clear boundaries and distinct cell bodies were included in the counts. The average value of five separate high-power fields in each section was used for measurements in 10 sections per brain. Cell density was indicated by the number of cells per square millimeter.

***Western blot***

The PAGs were dissected at the time of euthanasia and cryostored at −80 °C. The vlPAG region was harvested and ultrasonically treated with 400 μl of ice-cold lysis buffer and proteinase inhibitor supplementation. Protein concentrations were determined using the Bradford assay. Samples (20 μg of protein/lane) were electrophoresed on an SDS–polyacrylamide gel and then transferred to a polyvinylidene fluoride membrane. After transfer, the membrane was shaken for 1 hr in Tris-buffered saline (TBS) containing 0.1% Tween-20, 5% skim milk, and 0.2 % BSA, at room temperature (RT). Primary antibody staining (anti-P_2_X_3_, 1:400; anti-β-actin, 1:2000) was carried out at RT in TBS. Immunoreactive proteins were identified using chemiluminescent reagents. The relative optical density (ROD) of P_2_X_3_ was determined using the Quantity One software and normalized to β-actin. Immunoblot analysis was repeated at least three times to validate the data. The data were shown as a mean ratio of P_2_X_3_/ β-actin ± standard deviation (SD). 

***Experimental design***

The experiment included three series. In series 1, the time-course alterations were investigated in MWT & TWL after establishment of the osteocarcinoma pain model or multiple IP injections of neurotropin. According to the random number table, rats were divided into six groups (n=16/group): normal, sham-osteocarcinoma pain, osteocarcinoma pain, osteocarcinoma pain + low dose neurotropin (N_L_), osteocarcinoma pain+ medium-dose neurotropin (N_M_), and osteocarcinoma pain+ high dose neurotropin (N_H_) groups. Pain threshold values were measured in each group before osteocarcinoma pain surgery (day 0) and on days 5, 7, 10, 14, 18, and 21 after the surgery. Rats in N_L_, N_M_, and N_H_ groups were intraperitoneally injected with neurotropin 1 hr after completion of the pain threshold test at 6, 12, and 18 neurotropin units (NU)/kg daily for 7 consecutive days from the 15^th^ to the 21^st ^after the osteocarcinoma pain operation, in accordance with a previous report (23) and our preliminary results. 

In series 2, alterations of the P_2_X_3_ positive cell count and the P_2_X_3 _receptor protein levels in vlPAG were investigated. On the 21^st^ day after surgery, 16 rats/group in series 1 were euthanized, 8 rats/group was analyzed immunohistochemically, and the western blot was analyzed for the remaining 8 rats/group. 

In series 3, the analgesic effect of high-dose neurotropin (18 NU/kg) after A-317491 pretreatment was investigated. The selected dose of A-317491 for vlPAG microinjection was 1.5 nmol/0.3 µl according to prior reports (14, 24). On day 21 after surgery, 32 rats with osteocarcinoma pain were randomly classified into 4 groups, such as osteocarcinoma pain group, rats with osteocarcinoma pain with high dose neurotropin alone (osteocarcinoma pain+N_H_ group), rats with osteocarcinoma pain pretreated with A-317491 30 min prior to high dose neurotropin (osteocarcinoma pain+N_H_+A-317491 group), and rats with osteocarcinoma pain pretreated with sterile saline 30 min before high dose neurotropin (osteocarcinoma pain+N_H_+saline group). At 0, 15, 30, 45, 60, 75, and 90 min after being intraperitoneally injected with neurotropin, the antagonistic potency of A-317491 for the P_2_X_3_ receptor was assessed.

***Statistical analysis***

Data were analyzed using GraphPad Prism version 6.01. All data are expressed as mean ± SD. Behavioral endpoints were analyzed using the two-way analysis of variance (ANOVA) repeatedly. Other statistical analysis was performed with one-way ANOVA, and Tukey’s *post-hoc* analysis was carried out for multiple mean value comparisons between groups. The Student’s t-test was used for comparing these only two independent groups. Statistical significance was assigned for differences with a probability value of <0.05.

## Results

***Changes in pain threshold value by cancer cell inoculation***

**Figure 1:**
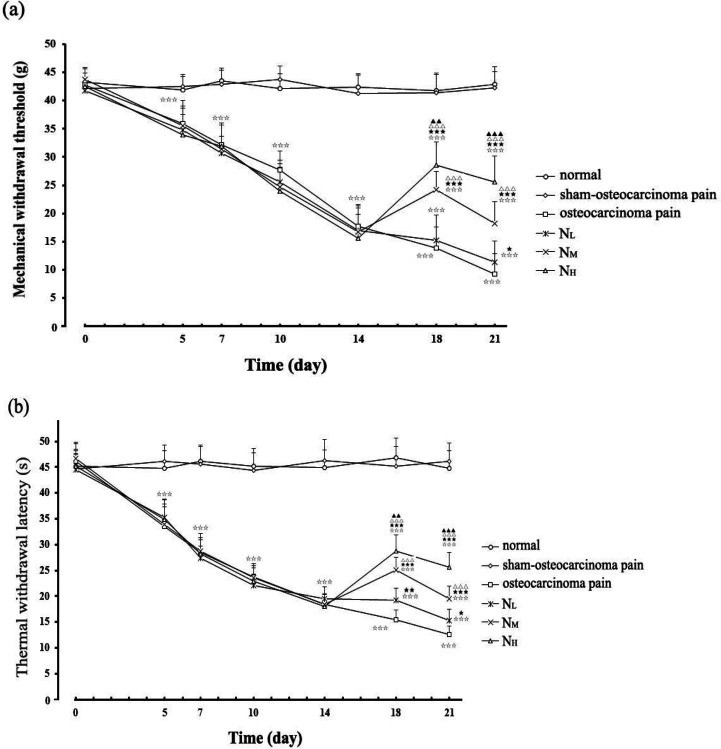
Changes in mechanical allodynia (a) and thermal hypersensitivity (b) after cancer cell inoculation or neurotropin injections. The MWT values of the osteocarcinoma pain group significantly decreased gradually from the 5th day to the 21st day after osteocarcinoma pain surgery. At all observation time points, MWT of the sham-osteocarcinoma pain group and the normal groups remained largely constant, with no statistically significant differences between groups. MWT increased after IP injection of neurotropin in NL, NM, and NH groups in a dose-dependent manner. The observed trend in TWL closely resembled the trend of MWT (n=16). At the same observation point, *P*<0.001 versus sham-osteocarcinoma pain group; *P*<0.05, *P*<0.01, *P*<0.001 versus osteocarcinoma pain group; *P*<0.001 versus NL group; *P*<0.001, P<0.001 versus NM group

No significant difference was found in preoperative mean MWT values among normal, sham-osteocarcinoma pain, and osteocarcinoma pain groups. The osteocarcinoma pain group had a progressive decrease in hind-paw MWT from 42.75 ±2.84 g before cancer cell inoculation to 27.63 ± 3.38 g on the 10^th^ day (*P*<0.001) and 9.26 ± 3.57 g on the 21^st^ day (*P*<0.001). The MWT values of the sham-osteocarcinoma pain group were stable from the 5^th^ day to the 21^st^ day after cancer cell inoculation with an insignificant difference between the sham-osteocarcinoma pain and normal control groups (*P*>0.05). The time-course alteration of TWL presented a similar trend to those in MWT (Figures 1a and 1b). 

***Evaluation of bone destruction in osteocarcinoma pain model by X-ray radiographs***

**Figure 2:**
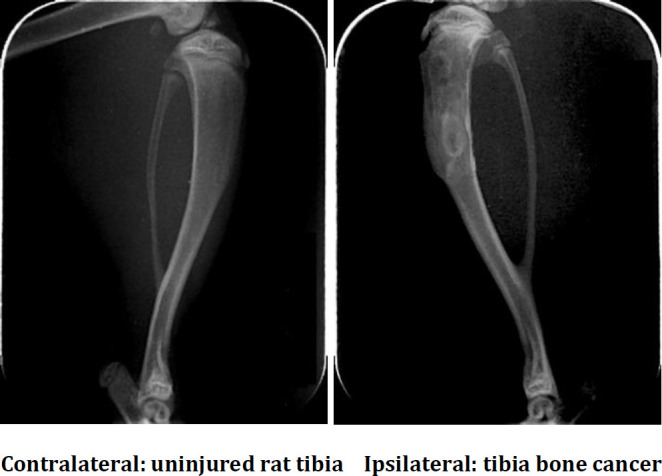
Tibia destruction after cancer cell injection. Radiography of the contralateral tibia shows an intact tibia, whereas the ipsilateral tibia shows irregular medullary cavity opacity and bone structure discontinuity proximal to the site of cancer cell injection

Radiological imaging was used to validate the osteocarcinoma pain model. On the 21^st^ day after inoculation of cancer cells, irregular medullary cavity opacity and bone structure discontinuity were observed in rat tibia inoculated with cancer cells (ipsilateral) via X-ray, suggesting bone destruction proximal to the site of SHZ-88 cell injection. On the contralateral tibia, no bone deficits were observed, suggesting a localized effect (Figure 2).

***Expression variation of P***_2_ ***X***_3_ ***receptor in the vlPAG after inoculation of cancerous cells***

**Figure 3:**
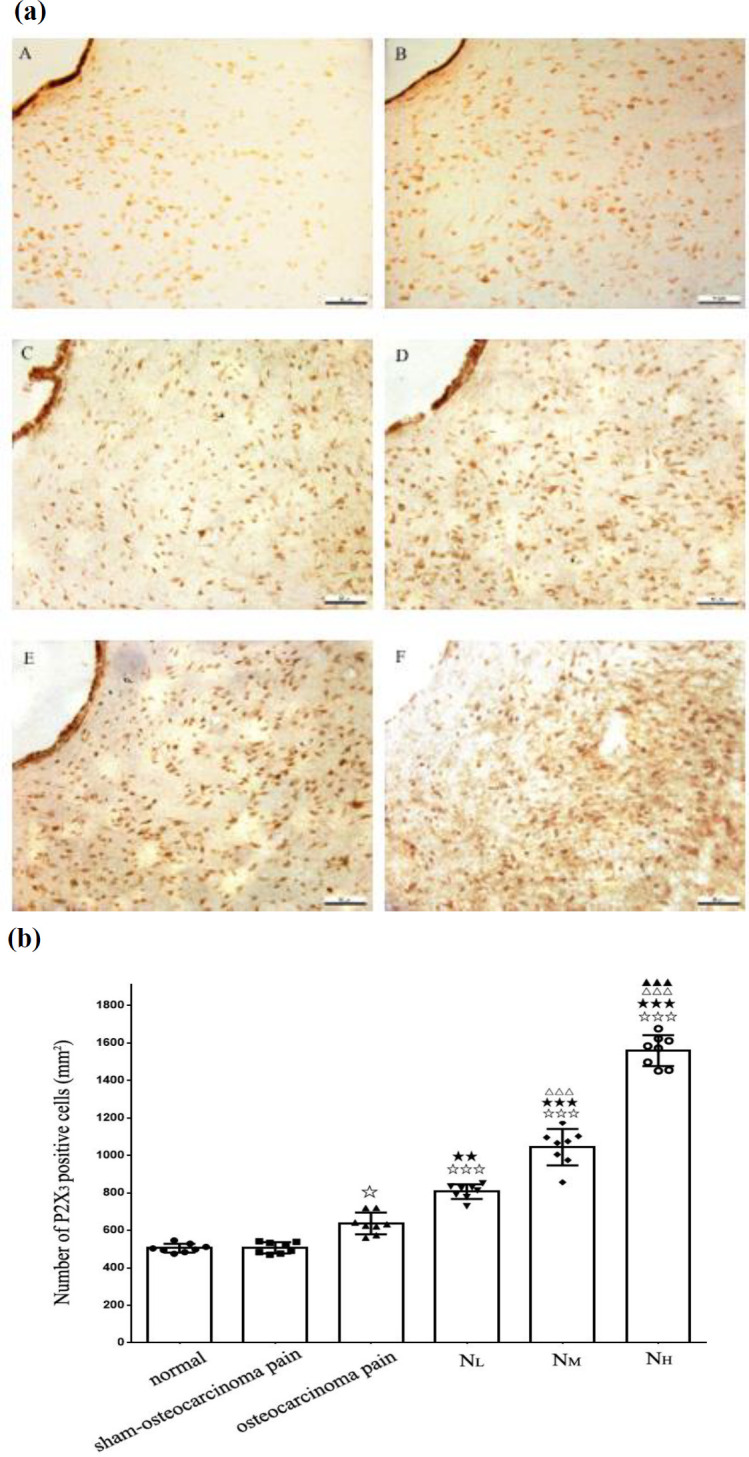
Changes in the expression of P2X3 receptor in vlPAG. (a) Representative photographs are exhibited for vlPAG P2X3 receptor immunostaining in the normal (Figure 3a A), sham-osteocarcinoma pain (Figure 3a B), osteocarcinoma pain (Figure 3a C), NL (Figure 3a D), NM (Figure 3a E), and NH groups (Figure 3a F). Scale bar = 50 μm. (b) The histogram demonstrates the number of vlPAG P2X3-positive cells. No obvious changes were observed in P2X3 expression in the vlPAG between the normal and sham-osteocarcinoma pain groups, but compared with that in the normal and sham-osteocarcinoma pain groups, P2X3 receptor positivity was significantly higher in the vlPAG of osteocarcinoma pain group on the 21st day. Neurotropin doses of 6, 12, and 18 NU/kg significantly increased the number of vlPAG

Immunohistochemical analysis of the P_2_X_3_ receptor in the vlPAG revealed a relatively sparse P_2_X_3_ receptor-specific immune response. P2X_3_-positive cell number in the normal group was 505.54±24.82/mm^2 ^and 507.33±31.05/mm^2^ in the sham-osteocarcinoma pain group (*P*>0.05, n=8). In the osteocarcinoma pain groups, compared with the normal group and sham-osteocarcinoma pain groups on the 21^st^ day after osteocarcinoma pain operation (636.67±62.92/mm^2^, *P*<0.05, n=8), immunoreactivity especially for P_2_X_3_ receptor was slightly increased. (Figures 3a and 3b). 

***P***_2_ ***X***_3_ ***receptor protein levels in vlPAG***

**Figure 4:**
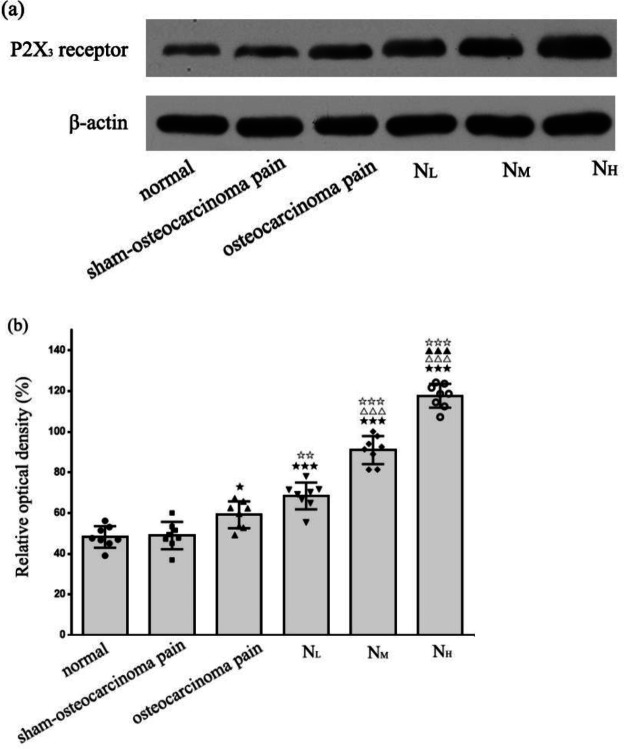
Alterations in P2X3 receptor protein content in vlPAG. (a) Immunoblot analysis detected a 55 kDa protein band, consistent with the molecular weight of the P2X3 receptor. (b) Levels of P2X3 receptor protein in each group. No significant differences in P2X3 receptor protein in vlPAG were observed between the normal and sham-osteocarcinoma pain groups, although expression mildly increased in the osteocarcinoma pain group. Neurotropin dosing of 6, 12, and 18 NU/kg significantly increased the P2X3 receptor protein expression levels in vlPAG. Compared with the normal group, n=8; *P*<0.05, *P*<0.001; Compared with osteocarcinoma pain group,*P*<0.01, *P*<0.001; Compared with NL group, *P*<0.001; Compared with NM group, *P*<0.001

In the normal, sham-osteocarcinoma pain, and osteocarcinoma pain groups, immunoblot analysis of vlPAG homogenates showed the presence of P_2_X_3_ receptor protein (55 kDa). An unobvious difference was found between the normal (ROD: 50.31 ± 4.11) and sham-osteocarcinoma pain groups (ROD: 50.08 ± 3.81, *P*>0.05). Through comparison between the normal and sham-osteocarcinoma pain groups, P_2_X_3 _protein expression was slightly higher with osteocarcinoma pain (ROD: 59.19 ± 4.18, *P*<0.05), (Figures 4a and 4b).

***Neurotropin treatment elevated pain threshold in osteocarcinoma pain rats***

Before intraperitoneal injection of neurotropin, no obvious difference in pain threshold was found among the osteocarcinoma pain, N_L_, N_M_, and N_H_ groups on the 14^th^ day after inoculation of cancer cells (*P*>0.05, n=16). Intraperitoneal injection of neurotropin at doses of 6, 12, and 18 NU/kg markedly increased MWT of N_L_, N_M_, and N_H _rats dose-dependently from the 18^th^ to the 21^st^ day after the inoculation of cancer cell. Furthermore, in the three neurotropin treatment groups, at the same time point, the N_L_ group had the lowest pain threshold values, whereas the N_H_ group had the highest, and the differences were statistically significant. Changes in TWL in response to neurotropin exhibited a similar pattern to MWT. (All *P*<0.001; *n *= 16; Figures 1a and 1b).

***Effects of different doses of neurotropin on P***_2_ ***X***_3_ ***protein levels in vlPAG of each group***

Neurotropin dosing of 6, 12, and 18 NU/kg once daily for 7 consecutive days led to significant up-regulation in vlPAG P_2_X_3_ receptor-positive cells and P_2_X_3 _receptor protein levels on day 21 after cancer cell injection (*P*<0.001, n=8; Figures 3a and 3b; Figures 4a and 4b). 

***Effect of A-317491 pretreatment on the analgesic effects of neurotropin intraperitoneal injection***

**Figure 5.**
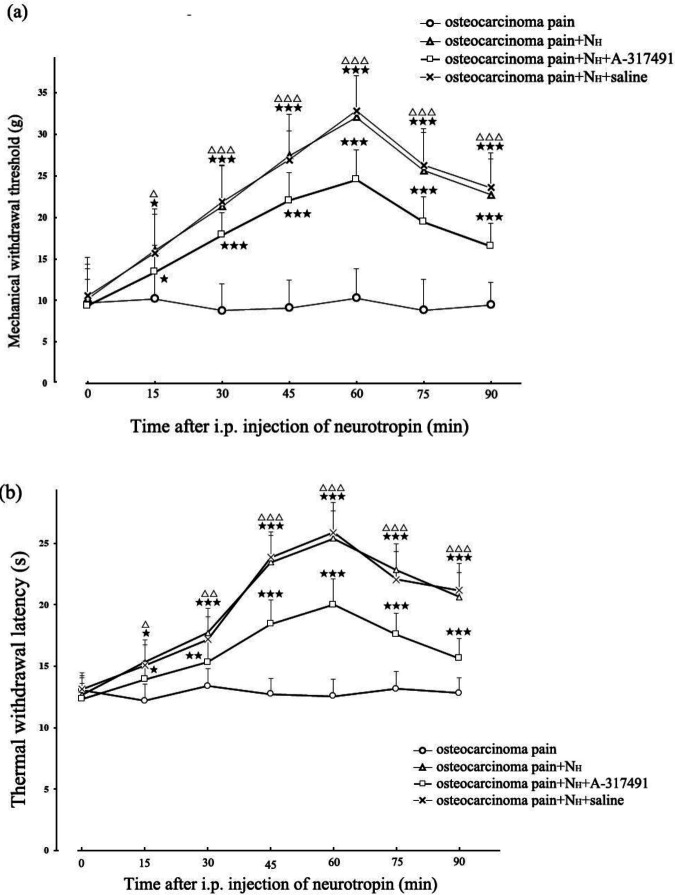
A-317491 attenuated the analgesic effect of 18 NU/kg neurotropin on osteocarcinoma pain. The pain threshold of osteocarcinoma pain in rats before vlPAG microinjection of A-317491 or IP injection of neurotropin served as the control (P>0.05 among all groups). Both MWT and TWL values were increased after IP injection of 18 NU/kg neurotropin, but intra-vlPAG injection with A-317491 attenuated the analgesic effect of neurotropin on osteocarcinoma pain. It was found that there was no significant difference between osteocarcinoma pain+NH and osteocarcinoma pain+NH+NS groups (n=8). Compared with osteocarcinoma pain group,*P*<0.05, *P*<0.01, and *P*<0.001; compared with osteocarcinoma pain+NH+A-317491 group, *P*<0.05,*P*<0.01, and*P*<0.001

MWT in the osteocarcinoma pain + N_H_ group increased from 10.20 ± 4.13 g to 32.15 ± 4.84 g at 60 min after IP injection of 18 NU/kg neurotropin. But the peak MWT response of the osteocarcinoma pain+N_H_+A-317491 group (24.55 ± 3.57 g) was lower than that in the osteocarcinoma pain + N_H _group essentially (*P*<0.001). The neurotropin’s antinociceptive effect was attenuated by A-317491 in vlPAG partially but significantly. Intra-vlPAG injection of an equivalent volume of sterile saline had no effect on the analgesic effect of IP neurotropin. Variation in TWL was similar to that of MWT. (Figures 5a and 5b).

## Discussion

The proximal medullary cavity of the tibia was inoculated with SHZ-88 breast cancer cells to establish a rat model with osteocarcinoma pain. P_2_X_3_ receptor expression was only mildly up-regulated in the vlPAG of the osteocarcinoma pain rats compared with that in normal and sham-osteocarcinoma pain groups. Neurotropin markedly ameliorated pain-associated behaviors in the osteocarcinoma pain rats, increased MWT & TWL values, and expression of increased P_2_X_3_ receptors in vlPAG of the osteocarcinoma pain rats with dependent dose. However, pre-treating with the selective P_2_X_3_ receptor antagonist A-317491 (1.5 nmol) via microinjection in vlPAG reversed the analgesic effects of high-dose neurotropin (18 NU/kg) partially and significantly. 

Neuropathic and inflammatory pains are the two major defining characteristics of cancer-induced pains. Notably, cancer-induced pain also has some unique aspects such as intense and persistent pain, along with aberrant psychological and behavioral symptoms (25, 26). Therefore, the mechanisms of cancer-induced pain are considerably more complex than any other type of chronic pain, making the analgesic treatment of this pain much more difficult from a clinical perspective. Bones are the third most prevalent site of malignant tumor metastasis, after the lungs and liver (27). Bone metastases are most commonly derived from multiple myeloma, breast, prostate, lungs, thyroid, kidneys, and ovaries cancers (28). Sites of metastases in the skeletal system are found in vertebrae with the most frequency, and then the pelvic bones, long bones (particularly the femur proximal to the primary tumor), and skull(29). In the present study, SHZ-88 breast cancer cells were injected into a single tibia of female SD rats. Consistent with a previous study (30), this method avoids widespread metastasis, and the rats maintained a relatively good physical condition throughout the entire study. Additionally, the maximum survival time of tumor cell-inoculated rats exceeded 50 days, allowing sufficient time to observe the changes in pain thresholds and evaluate the anti-nociceptive activity of neurotropin. 

P2X and P2Y receptors are widely distributed from peripheral subcutaneous tissues, dorsal root ganglions, and trigeminal ganglions to the central nervous system spinal dorsal horns and some supraspinal structures. At present, it is well-established that ATP and its receptors are regulating a very wide array of physiological and pathophysiological functions such as neurodevelopment, neurodegenerative diseases, cancer, osteoporosis, atherosclerosis, and pain (31, 32). ATP and/or other nucleotides are endogenous ligands of P2X and P2Y receptors and responsible for their pharmacological effects *in vivo*. One recent study found that intrathecal P_2_X_3_ antisense oligonucleotide treatment in a rat with Complete Freund’s Adjuvant-induced inflammatory pain decreased the P_2_X_3_ receptor expression in dorsal root ganglion and attenuated thermal allodynia (33), suggesting that activation of the P_2_X_3_ receptor in dorsal root ganglion is important in the development of pain. These findings were confirmed in another study that showed that intracerebroventricular (i.c.v.) administration of A-317491, a P_2_X_3_ receptor-selective antagonist, attenuated the antinociceptive effect produced by P2X receptor agonist alpha, beta-methylene ATP (α, β-meATP) in rats (34). It showed that P_2_X_3_ receptors of the cerebrospinal fluid-contacting nucleus and trigeminal subnucleus caudalis are important in mitigating inflammatory pain and masseter muscle pain processing via activation of PAG neurons (35, 36). Similar studies revealed that the P_2_X_3_ receptors in the thalamic ventromedial nucleus exhibit antinociceptive action in CFA-induced muscle pain in rats (24). These findings suggest that the P_2_X_3_ receptors at the supraspinal structure level likely play an inhibitory role in pain modulation. 

The midline structures of the brain constitute the endogenous analgesic system of the body. When this system is activated, it can inhibit incoming pain signals from the periphery and then manifest as an analgesic effect. In the present study, after peripheral pain information caused by osteocarcinoma pain was introduced into the central nervous system, the pain information was integrated, modulated, and then activated the cortex and other higher centers to promote the endogenous analgesic system. However, the degree of activation of the endogenous analgesic system was not sufficiently strong to counteract the intensity of incoming pain signals. As a result, the rats perceived pain. Intraperitoneal injection of neurotropin augmented P_2_X_3_ receptor activation in vlPAG and strengthened the endogenous analgesia system mediated by endogenous nucleotides. Accordingly, the body’s analgesic action suppressed or alleviated the pain information coming from the periphery. 

However, the mechanisms by which neurotropin promotes expression and activation of P_2_X_3_ have remained elusive. The P_2_X_3_ receptor is a purinergic receptor that can be activated by ATP and ATP analogs. In general, extracellular ATP is derived from exocytosis, and cellular membrane damage and mechanical stress can promote the release of ATP (37). Other recent studies have suggested that ATP can pass through the cell membrane via ATP-permeable channels (38). The activated neurons release ATP and use this molecule to send signals to other neurons or other cells in the brain (39). Additionally, ATP is a ubiquitous “glial transmitter” that is also released by astrocytes and other cells to activate purine receptors in neighboring cells (40). Therefore, P_2_X_3_ can be activated by the increase in the rate at which ATP is released due to the cell damage caused by peripheral tissue injury or inflammation. Simultaneously, intense activation of central neurons can increase ATP release and P_2_X_3_ receptor activation. 

Okai and colleagues (41) found that injection of neurotropin, i.c.v., increased the frequency of neuronal activation and firing in noradrenergic locus coeruleus (LC). This suggests that neurotropin has a direct excitatory effect on central neurons, and on this basis, the release of ATP from the excited neurons increased. In this study, the mechanism of activation of the P_2_X_3_ receptor by neurotropin may be related to the direct action of one of the components of neurotropin on vlPAG through the blood-brain barrier. 

Neurotropin is a well-known analgesic therapeutic extract used for neuropathic pain over the past 50 years (15, 42). Neurotropin also exerts neuroprotective effects against hypoxic-ischemic brain injury and Alzheimer’s disease by suppressing pro-inflammatory cytokines (43, 44). Despite its widespread use over many years, the complete chemical composition and analgesic properties of neurotropin are still unknown (45). By studying neuropathic pain in rats, Okazaki *et al*. (46) found that the analgesic mechanism of neurotropin includes activation of norepinergic and 5-HTergic down-regulation, as well as activation of 5-HT_3_ receptors in the spinal dorsal horn to strengthen the analgesic action of GABA interneurons. By using repeated cold water stress-induced muscle pain in rats, Nasu *et al. *(47) found that intramuscular injection of neurotropin alleviates mechanical pain sensitivity in the model rats, and further studies suggested that intrathecal injection of GABAergic, serotonergic and cholinergic receptor antagonists or intraperitoneal administration of opioid receptor antagonist attenuates the analgesic effects of neurotropin. Collectively these studies suggest that the analgesic effects of neurotropin on myalgia are mediated by several neurotransmitter systems. Neurotropin has also been shown to relieve neuropathic pain and depression-like behavior and related comorbidity in rats. That study’s authors determined that the neurotropin’s mechanisms are related to the of 5-HTergic neurons’ activation of the rostral ventromedial medulla and brain-derived neurotrophic factor’s release in the anterior cingulate cortex by neurotropin (42). Our results illustrate that expression of the P_2_X_3_ receptor was mildly increased in vlPAG of the rats with osteocarcinoma pain, and the expression was dose-dependently increased with repeated intraperitoneal injections of neurotropin. Owing to the elevated P_2_X_3_ receptor expression in vlPAG, MWT, and TWL in the rats with osteocarcinoma pain were up-regulated and the cancer-associated pain was relieved.

 A-317491 is a potent, selective P_2_X_3_ receptor antagonist with only very weak or no affinity for other receptors, channels, and enzymes (48). Additionally, the potency of A-317491 was observed for blockade of P_2_X_3_ receptor-mediated calcium influx; thus, A-317491 can block the functions of the P_2_X_3_ receptor (49). Several studies revealed that intrathecal and intraplantar administration of A-317491 is capable of relieving acute and chronic nociception in rat models (48, 50). In the present study, analgesic effects of neurotropin were weakened by vlPAG microinjection of A-317491 (1.5 nmol/0.3 μl), suggesting that the analgesic effect was at least partially activated by P_2_X_3_ receptors in vlPAG. As described in the previous section, activation of peripheral P_2_X_3_ receptors usually induces pain while reducing pain in supraspinal structures. These contradictory results further suggest that the role of the P_2_X_3_ receptor in supraspinal structures is different from spinal and peripheral regions. 

The present study is novel in that it has established the relationship between neurotropin and purine receptor expression, and further, it demonstrates that the purine signaling pathway is involved in the analgesic mechanisms of neurotropin. Thus, these findings further elucidate interactions between pain-related neurotransmitters. However, based on these findings, a follow-up study should be carried out that addresses the following: First, the exact location of where P_2_X_3_ receptors are expressed in vlPAG should be determined, namely the neurons in which they are expressed (NEergic, GABAergic, etc). Second, after activation of P_2_X_3_ receptors in vlPAG, the specific neural types and pathways by which the descending pain inhibition system is strengthened and acts on the spinal dorsal horn to produce an analgesic effect must be identified. Finally, given that neurotropin is an extract, the exact ingredients that are responsible for exerting its analgesic effects should be identified.

P2X3-positive cells in a dose-dependent manner. (n=8); *P*<0.05

and *P*<0.001 compared with sham-osteocarcinoma pain group

*P*<0.01 and *P*<0.001 versus osteocarcinoma pain group; *P*<0.001 versus NL group; *P*<0.001 compared with NM group

## Conclusion

Our study demonstrates that intraperitoneal injection of neurotropin promotes P_2_X_3_ purinergic receptor expression in vlPAG and that neurotropin dose-dependently exerts an analgesic effect on osteocarcinoma pain. Furthermore, analgesic effects of neurotropin are significantly reduced by intra-vlPAG injection of selective P2X_3_ receptor antagonist A-317491, suggesting that P_2_X_3_ receptor activation in vlPAG promotes endogenous pain inhibitory systems and is involved in mediating the analgesic effects of neurotropin. Overall, our findings provide evidence in support of the clinical application of neurotropin in osteocarcinoma pain, and that future pharmaceutical strategies targeting the P_2_X_3_ receptor may be highly efficacious in mitigating cancer-associated pain. 

## Authors’ Contributions

ZX Study conception and design; XFL and JXH Performing experiments; XFL and JXH Data analysis and draft manuscript preparation; ZX Critical revision of the paper; ZX Supervision of the research; XFL, JXH, and ZX Final approval of the version to be published.

## Conflicts of Interest

The authors declare that there are no potential interest conflicts in the authorship and/or publication of this paper herein.
